# Determination of specific proteins by the FIA principle

**DOI:** 10.1155/S1463924690000074

**Published:** 1990

**Authors:** Ib Andersen

**Affiliations:** 1 Clinical Chemistry Department Copenhagen County Hospital in Herlev Herlev DK 2730 Denmark; 2 Immuno Chemistry Department Novo-Nordisk A/S Sauntesvej 13 Gentofte DK 2820 Denmark

## Abstract

The following analytes have been investigated: urine albumin (u-albumin), plasma-transferrin (p-transferrin), p-haptoglobin, p-IgG, p-IgA, p-IgM, and p-orosomucoid. An unmodified commercial analytical system FIA Star (Tecator) with a two-channel injector (40 μl) was used. The prediluted plasma samples and antibodies are allowed to react for 33 s before the change in turbidity is measured as a Peak maximum at 405 nm. The optimal concentrations of calibrators and antibodies have been determined to secure antibody excess. Response time (i.e. delay between aspiration of a sample and presentation of the result in absorption units) is 75 s. Automatic print-out of the absorbance profile and movement of the sample rack further accounted for 21 s per sample, so the throughput is reduced to 75 determinations per 2 h. Results are available within an hour, compared to two-12 days with the present methods (electroimmunoassays). Parallel analyses with established methods/analysers show excellent agreement for u-albumin, p-transferrin and p-haptoglobin. For p-IgG, p-IgA and p-IgM the reaction time of 33 s is insufficient because their relative molecular masses (i.e. the size of the molecules) are so high, 150.000-971.000. Five minutes is a more adequate reaction time, which makes a serial analyser such as FIA Star unsuitable for larger workloads of samples of immunoglobulins. The plasma concentration of Orosomucoid is low, resulting in high sample blanks. It is therefore recommended that the reaction is followed kinetically if a serial analyser is used.


Journal of Automatic Chemistry, Vol. 12, No. 2 (March–April 1990), pp. 53–59

### Determination of specific proteins by the FIA principle

**Ib Andersen†**

*Clinical Chemistry Department, Copenhagen County Hospital in Herlev, DK 2730 Herlev, Denmark*

*The following analytes have been investigated: urine albumin (u-albumin), plasma-transferrin (p-transferrin), p-haptoglobin, p-IgG, p-IgA, p-IgM, and p-orosomucoid. An unmodified commercial analytical system FIA Star (Tecator) with a two-channel injector (40 μl) was used. The prediluted plasma samples and antibodies are allowed to react for 33 s before the change in turbidity is measured as a peak maximum at 405 nm. The optimal concentrations of calibrators and antibodies have been determined to secure antibody excess. Response time (i.e. delay between aspiration of a sample and presentation of the result in absorption units) is 75 s. Automatic print-out of the absorbance profile and movement of the sample rack further accounted for 21 s per sample, so the throughput is reduced to 75 determinations per 2 h. Results are available within an hour, compared to two–12 days with the present methods (electroimmunoassays). Parallel analyses with established methods/analysers show excellent agreement for u-albumin, p-transferrin and p-haptoglobin. For p-IgG, p-IgA and p-IgM the reaction time of 33 s is insufficient because their relative molecular masses (i.e. the size of the molecules) are so high, 150·000–971·000. Five minutes is a more adequate reaction time, which makes a serial analyser such as FIA Star unsuitable for larger workloads of samples of immunoglobulins. The plasma concentration of Orosomucoid is low, resulting in high sample blanks. It is therefore recommended that the reaction is followed kinetically if a serial analyser is used.*

### Introduction

In spite of the fact that clinical chemical applications account for 17% of the Flow Injection Analysis (FIA) literature [1], in practice the introduction of the FIA principle to clinical chemistry departments has been rather slow. This may be because of the potential for blood clots to block the system, especially where the sample material is blood, serum or plasma. On the other hand, chemical methods are so sensitive nowadays, that it is necessary to predilute the analyte many times to be within the linear range of the chemical methods. An obvious advantage is that many of the unwanted matrix effects caused by the sample material in clinical chemistry will disappear when diluting 10–20 times.

One of the most specific and sensitive chemical reactions is the 'immunoprecipitin reaction', i.e. the reaction between a protein (an antigen) and a specific antibody. The basis for the determination is the formation of aggregates when the bivalent antibody molecules combine with the usually multivalent antigen molecules [2]. In the state near equivalence, considerable cross-linking occurs. Antibody molecules bridge between antigen molecules to link several antigen molecules and many antibody molecules into large molecular aggregates, which form a precipitate. These aggregates, once they reach a molecular weight of about 3 million or greater, scatter light appreciably, and the scattering of light or the increasing absorbance (turbidity) is what is measured. The complex is soluble in an excess of antigen, so therefore it is important to check that the calibration curve increases within the biological concentration range, i.e. that antibody is always in surplus.

Polyethylenglycol, PEG (MWt: 6000), has been shown to accelerate the rate of formation of larger molecular aggregates at a concentration of 4%. If the antigen is charged, the PEG concentration should be increased to 7%, as is the case with the protein Orosomucoid. The reason for the catalytic effects of PEG is probably that it increases the concentration of the reactants in the reaction mixture, because it is both water-soluble and a large molecule in itself.

In 1972 the 'immunoprecipitin' reaction formed the basis of the work of Laurell [3], on how to determine the concentration of specific proteins by electroimmunoassay. Many clinical chemistry departments still use this method in spite of two serious drawbacks: it demands much manpower and it takes between two and 12 days before the results are available, depending on the number of re-runs and days between runs. The consumption of antibodies and antigens is small and is of minor importance in comparison to the long analytical time. Mechanization of the analytical process would be advantageous [4, 5]. It is a major investment (approximately $90 000) to buy a centrifugal analyser; so an FIA analyser was chosen to investigate the possibility of determining specific proteins, mainly because of the simplicity of the FIA principle, positive experience so far, and also because of the relatively minor investment (approximately $15 000).

### Reagents and materials

Carrier buffer: 0.05 mol/l phosphate, 0.1 mol/l sodium chloride, pH 7.4, with 4% PEG (7% PEG for Orosomucoid).
Dilution buffer for samples: the carrier buffer.
Dilution buffer for samples with very high concentration: the carrier buffer without PEG.

† Address for correspondence: I. Andersen, Immuno Chemistry Department, Novo-Nordisk A/S, Sauntesvej 13, DK 2820 Gentofte, Denmark.

0142–0453/90 $3.00 © 1990 Taylor & Francis Ltd.

*Table 1. Calibrator values, concentrations, normal ranges and maximum concentrations tested (found)† for antibody excess.*

| Quantity | Calibrator 1 | 2 | 3 | 4 | 5 | 6 | Units | Normal ranges | Maximum concentration tested (found) for antibody excess |
|---|---|---|---|---|---|---|---|---|---|
| U-albumin | 4·04 | 2·16 | 0·92 | 0·41 | 0·21 | | μmol/l | 0·03–0·5 | 9·00‡ |
| P-transferrin | 98·4 | 79·2 | 59·8 | 40·1 | 20·7 | | μmol/l | 42–72 | 205 |
| P-haptoglobin | 1·02 | 0·73 | 0·49 | 0·25 | | | μmol/l | 5–46 | 80 |
| P-IgG | 0·099 | 0·080 | 0·060 | 0·040 | 0·020 | | g/l | 6·82–15·70 | 40·30 |
| P-IgA | 0·096 | 0·077 | 0·059 | 0·039 | 0·020 | | g/l | 0·56– 3·30 | 9·30 |
| P-IgM | 0·32 | 0·24 | 0·16 | 0·075 | | | g/l | 0·18– 1·29 | (7·08) |
| P-orosomucoid | 5·06 | 2.95 | 1·61 | 0·84 | 0·64 | 0·43 | μmol/l | 11–30 | (105) |

† The highest levels found in plasma in years of experience.
‡ Because the biological range is so wide, 0–250 μmol/l, the urine concentration is roughly estimated by means of an 'Albustix'. Depending on the result the sample is prediluted with p-buffer, pH 7·4 (0, 10 or 50 times) to a concentration which is in the range of albumin calibrator 4 or 5. The maximum concentration after predilution is 5·05 μmol/l. So albumin antibody will always be in excess, when predilution is performed as described.

*Table 2. Specific proteins.*

| Quantity | Molecular weight | Sample material | Predilution of the sample (times) | Dilution of the antibody (times) | Antibody producer |
|---|---|---|---|---|---|
| U-albumin | 66·290 | Urine | 6 | 15 | Dakopatts, A119 |
| P-transferrin | 76·500 | Plasma† | 51 | 11 | Dakopatts, A061<br>Atlantic antibodies 016-11 |
| P-haptoglobin | 86·018 | — | 51 | 11 | Dakopatts, A030 |
| P-Ig-G | 150·000 | — | 201 | 11 | Dakopatts, A090 |
| P-Ig-A | 160·000 | — | 101 | 11 | Dakopatts, Q332, A092<br>Atlantic antibodies 011-11 |
| P-Ig-M | 971·000 | — | 5 | 11 | Dakopatts, A091 |
| P-orosomucoid | 41·000 | — | 11‡ | 11 | Dakopatts, A011 |

† Concentration of the anticoagulant $EDTAK_2$ is 7·2 mmol/l.
‡ The predilution p-buffer differs from the one generally used: 7% PEG instead of 4% PEG.

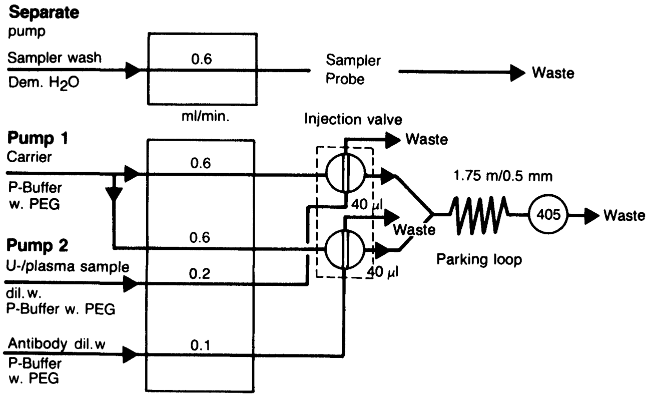

*Figure 1. Manifold diagram for FIA specific proteins. Carrier is p-buffer with 4% PEG. The proteins were u-albumin, p-transferrin, p-haptoglobin, p-IgG, p-IgA, p-IgM and p-orosomucoid.*

Dilution buffer for antibodies: the carrier buffer.

Polyethylenglycol, PEG (MWt: 6000), analytical grade, Merck.

Range of calibration solutions: see table 1.

Antibodies used: see table 2.

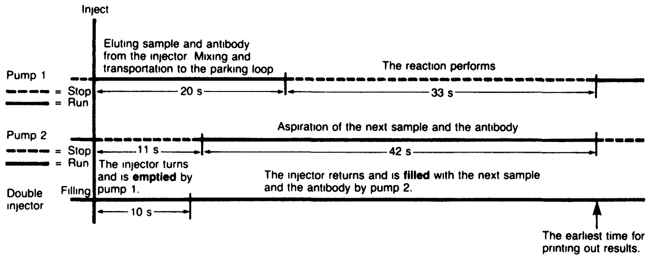

*Figure 2. The sequences of the pumps and the double injector movements. As soon as the reaction time (33 s) has elapsed pump 1 starts transporting the reaction products through the flowcell. The profile and the maximum absorbance measured at 405 nm is printed out.*

Calibrator: Cal DSKK 8412 (from Danish Society of Clinical Chemistry).

Plasma samples: anticoagulant—dipotassium EDTA, 7·2 mmol/l.

### Methods

In references [6], [7] and [8] examples are given of how to determine human IgG by means of Goat anti-human IgG antiserum. Based on these experiments, the following

The calibration function, $y = a + bx + cx^2$, has been calculated in connection with the daily run of patient samples through a number of days.
The fit of the data points (x,y) = (calibrator conc., mE at 405 nm) to the calibration function is estimated by means of the coefficient of correlation, R.

| Quantity | Number of Determinations | $\bar{R}$ | Range of R |
|---|---|---|---|
| Transferrin | 11 | 0.9991 | 0.9975 - 0.9997 |
| Haptoglobin | 11 | 0.9992 | 0.9975 - 0.9999 |
| IgG | 5 | 0.9982 | 0.9953 - 0.9996 |
| IgA | 2 | 0.9995 | 0.9992 - 0.9997 |

*Figure 3. Calibration function. Reproducibility.*

attempts have been made to determine specific proteins in plasma (urine) on an unmodified FIA analyser, FIA Star (Tecator).

The following seven specific proteins were investigated:

u-albumin, p-transferrin, p-haptoglobin, p-IgG, p-IgA, p-IgM and p-orosomucoid.

From Price *et al.* [5], it is known that the immunoprecipitin reaction does not occur immediately but the main part of the reaction takes place within the first minutes after mixing and, in practice, the reaction has finished after 5 minutes.

Most other chemical reactions occur immediately, so the limiting factor for the throughput is the transportation process to and from the detection cell. To combine the necessary transportation of diluted plasma material from the sampler to the sampling loop with a prolonged reaction time, the set up described in figure 1 was chosen, see figure 1. Before injection the sample and antibody loops are filled by means of pump 2. When injections occur the double injector valve turns, pump 2 stops and pump 1 starts eluting diluted sample and antibody separately from the injector. After 10 s the loops of the injector are emptied by pump 1, and the injector returns to the filling position ready for the next diluted sample and for antibody reagent, and pump 2 starts immediately after. Pump 1 continues for 20 s, transporting the reactants through the 'CHEMIFOLD', where they are mixed, to a parking loop of 1·75 m, and then pump 1 stops. Now the 'Precipitin' reaction is allowed to proceed for 33 s, while pump 2 is filling the sample and antibody loops. When the 33 s has elapsed, pump 1 starts transporting the reaction products through the flowcell. At the same time pump 2 stops. As soon as the maximum of the absorbance profile is determined at 405 nm (which is the shortest recommended wavelength) it is printed-out together with the absorbance profile and the sequence number. Injection of the next sample and antibody takes place, when the double injector valve turns and the sequences described above are repeated. Figure 2 shows a schematic overview of the functions.

Because of the combination of aspirating the next sample, while the former is still in the analyser, it has been impossible to use the built-in calibration option. Therefore the coefficients of the calibration function have been calculated separately for each quantity. The fit of the datapoints to the function is estimated by means of the coefficient of correlation, *r* (see figure 3). A series of patient samples is always preceded and succeeded by calibration solutions, the number of which varies between four and six, see table 1. For each of the seven proteins investigated, the necessary sample and antibody dilution, which gives a reproducible signal is determined, i.e. turbidity at 405 nm, in a set-up according to figure 1 (see table 2). The throughput is then nearly 75 determinations per 2 h, and the response time is 75 s. No drift has been observed through 2 h of unattended analysis, neither concerning the blank's signal nor that of the calibrators.

### Results

For applications in a clinical laboratory it would be attractive if the only difference between the analytes were the antibody used and the predilution of the samples, thereby reducing the need for different parameter settings of the analyser. This, and the prevention of sample clotting, were considered important.

Originally, the FIA method for U-albumin was compared to an immunoprecipitin method performed on a discrete analyser (Reaction Rate Analyzer, LKB 8600),

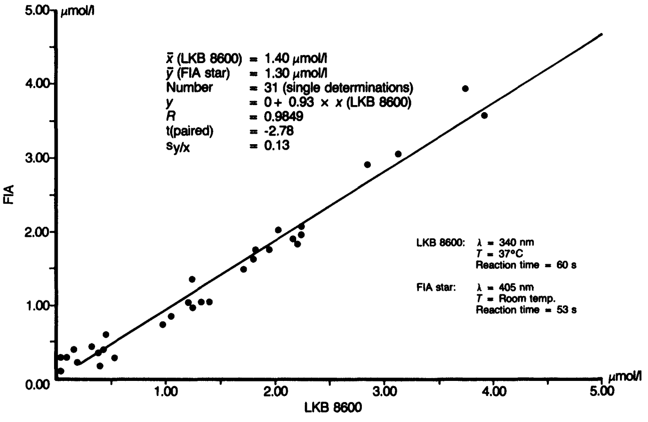

*Figure 4. U-albumin, concentration. Parallel analysis (LKB 8600 versus FIA Star). The immunoprecipitation methods are identical concerning the concentration of reactants.*

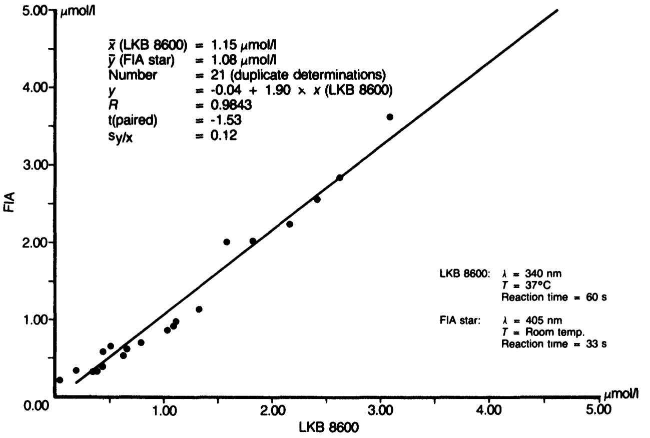

*Figure 5. U-albumin, concentration. Parallel analysis (LKB 8600 versus FIA Star). The immunoprecipitation methods are identical concerning the concentration of reactants.*

*Table 3. Determination of the reproducibility within series of u-albumin, p-transferrin and p-haptoglobin at three different levels of concentration.*

| Component | Concen-tration | Unit | Level | Number | Absorbance (× $10^{-3}$) Mean | SD | CV (%) |
|---|---|---|---|---|---|---|---|
| U-albumin | 3·8 | μmol/l | High | 23 | 75·4 | 4·9 | 6·5 |
| — | 2·5 | — | Medium | 24 | 63·5 | 2·4 | 3·7 |
| — | 0·9 | — | Low | 24 | 29·4 | 2·0 | 6·9 |
| | | | | | Concentration (μmol/l) | | |
| P-transferrin | | | High | 14 | 69·1 | 2·7 | 3·9 |
| — | | | Medium | 14 | 52·9 | 2·0 | 3·8 |
| — | | | Low | 14 | 36·0 | 1·4 | 3·8 |
| P-haptoglobin | | | High | 16 | 34·5 | 1·08 | 3·1 |
| — | | | Medium | 16 | 29·6 | 0·56 | 1·9 |
| — | | | Low | 16 | 13·3 | 0·48 | 3·6 |

*Table 4. Determination of the reproducibility within series of p-IgG and p-IgA at three different levels of concentration.*

| Component | Level | Number | Concentration (g/l) Mean | SD | CV (%) |
|---|---|---|---|---|---|
| P-IgG | High | 12 | 11·7 | 0·6 | 4·8 |
| — | Medium | 12 | 9·7 | 0·3 | 3·2 |
| — | Low | 12 | 7·1 | 0·5 | 7·7 |
| P-IgA | High | 14 | 4·38 | 0·088 | 2·0 |
| — | Medium | 14 | 2·02 | 0·047 | 2·3 |
| — | Low | 14 | 1·23 | 0·039 | 3·1 |

where the concentrations of albumin and anti-albumin were identical in the two set-ups. Agreements of the results seemed so promising (figure 4) that the reaction time was shortened from 53 s to 33 s to see if the accordance was still satisfactory (figure 5). The set-up from that experiment was also used for the other investigated proteins. Besides parallel analysis with the routine method, which, for all analytes, except albumin, is the Laurell method [3], the reproducibility (standard deviation, SD) and coefficient of variation (CV) within series was determined several times at three levels within the biological range:

For albumin, see figure 5 and table 3
For transferrin, see figure 6 and table 3
For haptoglobin, see figure 7 and table 3
For IgG, see figure 8 and table 4
For IgA, see figure 9 and table 4

Parallel analysis for IgA (see figure 9) showed a poor correlation, $r = 0{\cdot}65$, when compared to the routine method. Therefore the same samples were also analysed on a Cobas Bio Centrifugal analyser, according to Price *et al.* [5]. This analyser measures the turbidity when the reaction is complete after 5–10 min. The coefficient of correlation, $r$, was 0·93 (see figure 9). As can be seen especially at high concentration of IgA (>2·80 g/l), some patient samples do react more slowly than would have been expected. The accuracy of the FIA determination is therefore not satisfactory, although the reproducibility is (see table 4).

The parallel analysis for Ig-M (see figure 10) shows a fair agreement between the two methods at low concentrations but marked deviations at higher concentrations (1·60–4·00 g/l).

To investigate the reason for these differences, nine of the sample were analysed on a Reaction Rate Analyzer (LKB

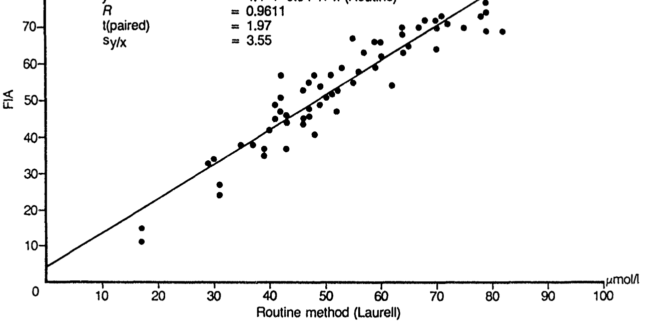

*Figure 6. P-transferrin, concentration. Parallel analysis (routine method versus FIA Star).*

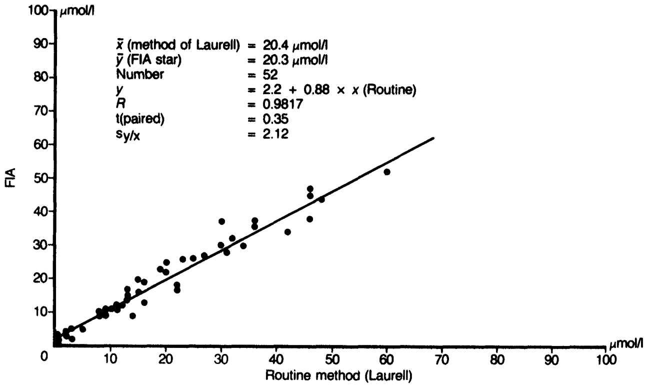

*Figure 7. P-haptoglobin, concentration. Parallel analysis (routine method versus FIA Star).*

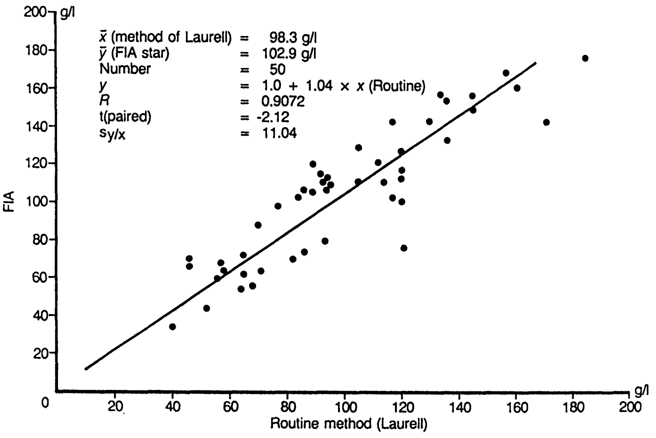

*Figure 8. P-IgG, concentration. Parallel analysis (routine method versus FIA Star).*

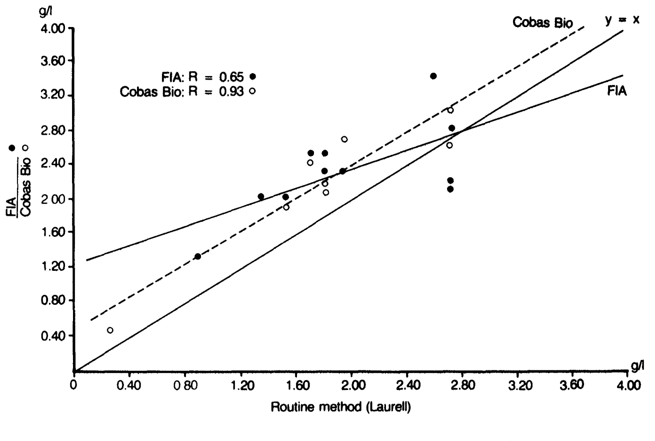

*Figure 9. P-IgA, concentration. Parallel analysis (routine method versus FIA Star), see closed circles. Parallel analysis (routine method versus Cobas Bio), see open circles.*

8600). The sample and antibody dilutions were identical in the two set-ups. The increase in absorbance with time was registered on a recorder connected to the LKB 8600 and the measured absorbance after 33 s was compared to the absorbance after 5 min, A(33 s)/A(300 s). This ratio was plotted against the difference between routine and FIA results (see figure 11). It is seen that the differences increase with falling ratio. The reaction is far from completion after 33 s for high differences; on average, the reactions were completed after 4·3 min—the maximum value found was 8·6 min. Typically, the absorbance increases exponentially with time within the first minute after mixing. The final absorbance is reached asymptotically.

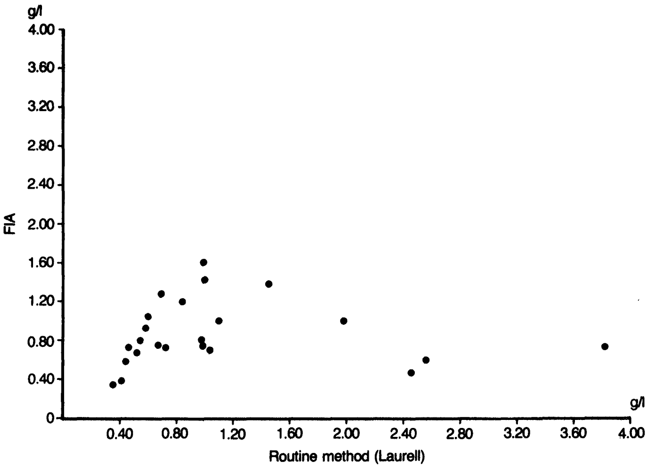

*Figure 10. P-IgM, concentration. Parallel analysis (routine method versus FIA Star).*

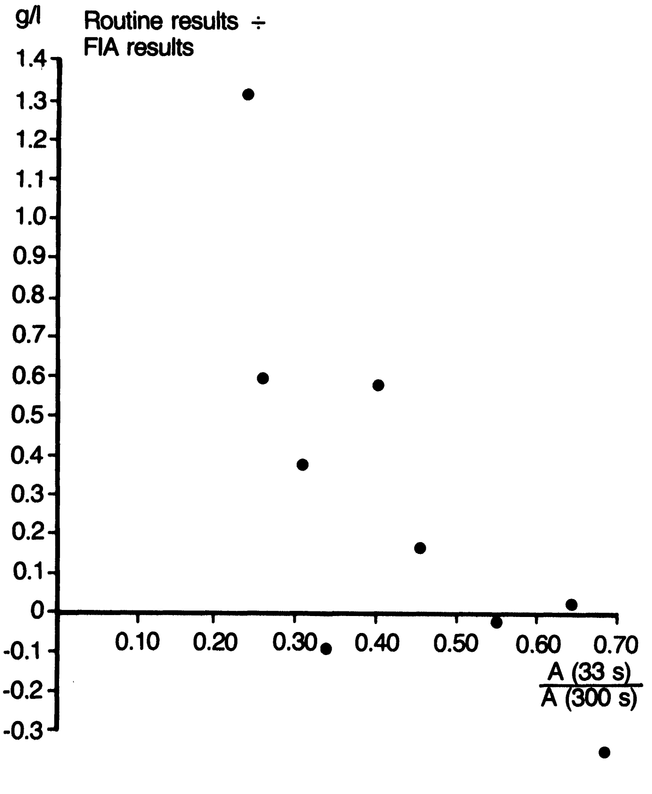

*Figure 11. P-IgM, concentration. Differences between routine and FIA results as a function of completion of the reaction. The increase in 405 absorbance with time is registered on a recorder connected to LKB 8600 (reaction rate analyser). Concentrations of samples and antibody are identical with the FIA Star set-up. The absorbance after 33 s is compared with the absorbance after 5 min, A(33 s)/A(300 s).*

When trying to optimize the analytical conditions for the protein p-orosomucoid two problems were encountered (see figure 12). First, the plasma can only be diluted 11 times to obtain a suitable absorbance signal, i.e. an immuno-complex of reasonable size within the 33 s of reaction time. Therefore the sample blank becomes so high that the series has to be rerun, using p-buffer as reagent, to determine the blank value. Secondly, the absorbance of the highest calibrator is less than the lowest calibrator. The reason can only be that here the antigen is in excess in relation to the amount of antibody present. It is a consequence of the biphasic form of the precipitin reaction curve, which has an absorbance maximum between the regions of antibody and antigen excess. All samples, therefore, have to be prediluted to be sure that their concentrations are in the range of antibody excess. For orosomucoid the maximum concentration found in plasma is 105 µmol/l. Figure 12 shows that the absorbance maximum is at a concentration of 3·0 µmol/l, so the predilution factor has to be 105/3·0 to ensure that the calibration curve is ascending. From a practical point of view this is not a satisfactory procedure.

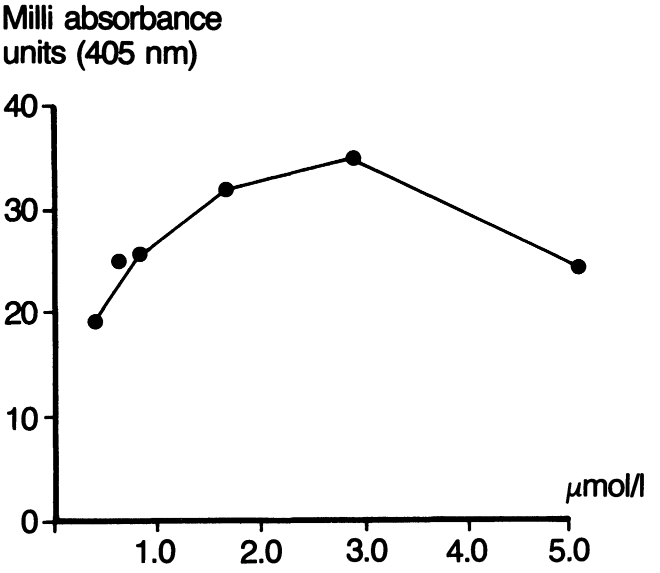

*Figure 12. P-orosomucoid, concentration. Calibration curve. Where x-axis: diluted standard solutions; y-axis: absorbance difference between sample and sample blank.*

### Discussion

*U-albumin*

The high coefficient of correlation, $r = 0{\cdot}9849$ (see figure 4), between the routine method and the FIA method, together with the slope (Deming correlation line) of 0·93 proves the good agreement between the results of the two methods. A t-test (paired) = −2·78 shows that there is a difference on the results at the 5% significance level. A coefficient of correlation of the same order, $r = 0{\cdot}9843$, was calculated from a new set of results after having shortened the reaction time from 53 s to 33 s (see figure 5). A t-test (paired) at 1·53 shows that there is no difference on the results at the 5% significance level. This proves that the main part of the reaction has occurred within 33 s of mixing. Therefore the other investigations were performed with this set-up. The reproducibilities within series at three different levels are shown in table 3. The coefficient of variation is satisfactory in comparison with the results obtained by Rifai *et al.* [9].

*P-transferrin*

Parallel analysis was performed using the routine Laurell method and the FIA method, (see figure 6). Good agreement was found between the mean of the samples determined by the routine method, calculated as 56·0 µmol/l and the mean of the results by the FIA method, 57·1 µmol/l. The coefficient of correlation, $r$, was calculated as 0·9611. A t-test (paired) at 1·97 shows that there is no difference on the results at the 5% significance level. As for u-albumin the reproducibility within series for p-transferrin (see table 4) was satisfactory.

*P-haptoglobin*

Figure 7 shows the results of parallel analysis between the routine (Laurell) method and the FIA method on 52 patient samples. A t-test (paired) at 0·35 shows that there is no difference on the results at the 5% significance level. The coefficient of correlation, $r$, was 0·9817. The reproducibility within series (see table 5), varies from 1·9 to 3·6%. The results were considered satisfactory.

*P-IgG*

Figure 8 shows the result of parallel analysis between the routine (Laurell) method and the FIA method. The slope of the Deming regression line and the agreements of the means are satisfactory, but the coefficient of correlation, $r$, is only 0·9072, which is *not* satisfactory. According to Penberthy [10], $r^2$ is a measure of the variance between the two methods, and $(1 - r^2)$ is due to other sources of variance. In this case $(1 - r^2)$ is 0·19, that is 19%. When one considers that the molecular weight of an antigen IgG molecule is nearly 150 000, the reason for the poor agreement between the two methods is that the main part of the precipitin reaction has not occurred after 33 s, which is the reaction time in this FIA set-up. Because of the timing of the FIA principle, the reproducibility is good (see table 4), although the correlation is not satisfactory.

*P-IgA*

The immunoglobulins (G, A and M) have relative molecular masses which are significantly higher than the other proteins investigated (see table 2): 150 000 for IgG, more than 160 000 for IgA and more than 971 000 for IgM. The problems encountered for p-IgG could therefore be expected for IgA and IgM. As well as parallel analyses between the routine (Laurell) method and the FIA method, eight samples were also analysed on a centrifugal analyser (Cobas Bio) used in endpoint mode. The reaction was an antigen–antibody reaction [4, 5].

As can be seen from figure 9, the correlation coefficient, $r$, between the routine (Laurell) method and the Cobas Bio method is far better, 0·93 versus 0·65, than for the FIA method. Therefore, if a better agreement between the present routine method and the FIA method is to be obtained, the reaction time must be increased considerably.

The maximum time which can be chosen in FIA Star is 99 s. Whether this is enough to give a satisfactory coefficient of correlation with the present routine method was not tested. This is because the requests for IgG, IgA and IgM are so numerous that the daily analysis time for determining these quantities would be unduly long and it would not be advantageous to change to the FIA Star analyser. The reproducibility is satisfactory for IgA (see table 4).

*P-IgM*

Parallel analysis between the routine method and the FIA method was performed for p-IgM. From figure 10 it can be seen that at high concentrations, low FIA results were found. The reaction was also followed for 300 s for nine different samples on the recorder of a Reaction Rate Analyzer (LKB 8600) at 405 nm. The absorbances were measured 33 s and 300 s after mixing. The ratio of the absorbances is plotted against the differences between routine and FIA results in figure 11. The differences increase as the absorbance ratio decreases. The ratio is an expression of how far the reaction is from completion. The only explanation is that 33 s is too short a time for the huge antigen and the polyclonal antibody molecules to form a complex of sufficient size to scatter light.

*P-orosomucoid*

Figure 12 shows the calibration curve. Because of the biphasic course, a high sample antigen concentration can achieve a lower absorbance at 405 nm than a sample with a lower antigen concentration, and it is therefore necessary to predilute all plasma samples 35 times to be sure that the concentration is less than the critical value of 3·0 µmol/l. Within this range the calibration curve is ascending see the final part of the 'Results' section).

The absorbance of the sample itself (the sample blank) is relatively high because of the low predilution factor of 11. This means that it is necessary to run the sample series without antibody in the p-buffer to obtain a sample blank value for each sample. It is therefore not practical to use *this* particular FIA set up in determining p-orosomucoid.†

† After the investigations were complete I was informed that the FIA Star system is also able to measure differences in absorbances between preset times. By stopping the flow when the reaction has just started (and the reaction mixture is in the flow-through cell), the initial absorbance can be measured and also the absorbance after, for example, 33 s).

### Conclusion

The FIA-method set-up as described in figure 1 can be used for analysing u-albumin, p-transferrin and p-haptoglobin.

For IgG, IgA and IgM FIA Star is not suitable because of the much longer reaction time of 5–10 min.

For p-orosomucoid the FIA principle can be used if the FIA analyser measures the reaction kinetically, so the sample blank determination is avoided; however, the predilution of the plasma sample must be sufficient (35 times) to ensure that the calibration curve is ascending. The kinetic set-up can very easily be applied to the system described in this work. The incubation of the reaction mixture can take place in the flow cell instead of in a delay loop.

Clotting has occurred two or three times in the aspiration tube because of a fibrin clot in the prediluted sample. The prediluted samples had at that time been kept overnight in the refrigerator. The problem was solved by using freshly diluted EDTA plasma.

### Acknowledgement

The author is grateful to the audio-visual department of the Copenhagen County Hospitals for assistance in preparing figures, and to Erna Quist for typing the manuscript.

Dr Mogens Sandbjerg Hansen is especially thanked for his critical revision of the English manuscript; and thanks are also due to Professor Jaromir Ružička and Professor Ole Siggaard-Andersen for their valuable comments on the manuscript.

### References

1. Ružička, J. and Hansen, E. H., *Analytica Chimica Acta*, **179** (1986), 1.
2. Stenberg, J. C., *International Clinical Product Review*, **3** (1984), 16.
3. Laurell, C.-B., *Scandinavian Journal of Clinical Laboratory Investigation*, **29** (1972), 124.
4. Price, C. P. *et al.*, *Centrifugal Analysers in Clinical Chemistry*, (Praeger Publishers, 1980), 449.
5. Price, C. P. *et al.*, *Centrifugal Analysers in Clinical Chemistry*, (Praeger Publishers, 1980), 477.
6. Hughes, A. *et al.*, *Analytical Proceedings*, **22** (1985), 16.
7. Worsfold, P. J., *Analytical Proceedings*, **22** (1985), 357.
8. Worsfold, P. J., *Analytica Chimica Acta*, **180** (1986), 56.
9. Rifai, N. *et al.*, *Clinical Biochemistry*, **20** (1987), 179.
10. Penberthy, L., *Clinical Biochemistry Reviews*, **7** (1986), 39.

